# 간호사의 병원 조기대응팀에 대한 인식과 만족도가 응급상황 관련 소진에 미치는 영향: 횡단적 연구

**DOI:** 10.7586/jkbns.25.023

**Published:** 2025-05-28

**Authors:** Bumin Kim, Nahyun Kim

**Affiliations:** 1 Department of Nursing, Graduate School of Keimyung University·Keimyung University Dongsan Hospital, Daegu, Korea; 1 계명대학교 대학원 간호학과·계명대학교 동산병원; 2 College of Nursing, Keimyung University, Daegu, Korea; 2 계명대학교 간호대학

**Keywords:** Hospital rapid response team, Perception, Job satisfaction, Burnout, professional, 조기대응팀, 인식, 만족도, 소진

## Abstract

**Purpose:**

The purpose of this descriptive cross-sectional study was to identify the effects of nurses' perceptions and satisfaction with the hospital rapid response team (RRT) on burnout related to emergency situations in Korea.

**Methods:**

The participants of the study were 277 nurses who had worked for more than six months in general wards at a tertiary hospital in Daegu City. Data were collected using a structured questionnaire from September 22, 2023 to October 2, 2023.

**Results:**

Burnout related to emergency situations showed a significant negative correlation with the perception of the RRT (r = −.24, *p* < .001) and satisfaction with the RRT (r = −.16, *p* = .008). Perception and satisfaction regarding the RRT (r = .65, *p* < .001) had a significant positive correlation. In addition to general characteristics such as education level (β = −.24, *p* = .038), clinical experience (β = −.16, *p* = .007), work type (β = .19, *p* = .016), and job experience level (β = −.26, *p* < .001), perception of the RRT (β = −.16, *p* = .006) was found to affect burnout related to emergency situations. Multiple regression analysis showed that the regression model was appropriate (F = 2.07, *p* < .001), with an explanatory power of approximately 15%.

**Conclusion:**

To reduce burnout related to emergency situations, it is necessary to foster positive perceptions of the RRT.

## 서론

### 1. 연구의 필요성

조기대응팀(rapid response team, RRT)은 일반병동에서 급성으로 상태가 악화되는 환자를 조기에 발견하고, 신속한 평가와 치료를 제공하는 전문 의료지원팀이다. RRT의 주요 목적은 비계획적인 중환자실 입실, 심정지, 사망 등의 중대한 사건을 예방하는 데 있다[1]. RRT는 중환자 치료에 대한 전문 훈련을 받은 의사와 간호사로 구성되며, 이 중 RRT 전담 간호사는 병동 환자에 대한 지속적인 모니터링을 통해 조기 개입의 핵심적인 역할을 수행한다[2].

환자의 급속한 상태악화 및 심각한 질병 발생위험은 생리적인 지표 변화를 통해 감지할 수 있으며[3] 환자의 상태가 악화되기 약 8∼48시간 전 비정상적인 활력징후가 나타난다[4]. 그러나 병동간호사는 과중한 업무량으로 환자의 상태가 악화되는 것을 즉각적으로 인식하기가 어렵고, 응급실이나 중환자실에 비해 일반병동은 중증 환자 치료에 대해 훈련받은 의료진이 부족하기 때문에 급성 악화 환자에 대한 조기 발견 및 신속 대응에 한계가 있다[5]. 특히 급·만성 질환을 지닌 환자에게 발생하는 예기치 못한 응급상황은 스트레스를 가중시키고 이것이 반복되거나 지속될 경우 간호사는 높은 소진을 경험하게 된다[6]. 또한 응급상황에서의 높은 간호 요구도 및 간호 의존도는 간호사로 하여금 괴로움, 압도감, 자기 회의, 극도의 불안을 야기해 환자에 대한 관심 회피, 업무에 대한 부정적인 인식으로 이어져 이직 및 사직의 주요 원인이 된다[7,8]. 지속되는 응급상황 관련 소진은 직무 만족도 저하, 이직률 증가, 장기적으로 양질의 간호 서비스 제공 및 환자 안전에 치명적인 위협을 초래할 수 있다[7]. 선행연구에 따르면 응급상황 관련 소진은 개인적 요인, 조직적 요인, 환경적 요인 등 다양한 요인의 영향을 받는다[6]. 경험이 부족한 간호사는 응급상황에서 더 큰 스트레스를 경험하며, 교대근무로 인한 수면부족과 피로 누적은 소진을 가중시키는 요인으로 작용하기에 적극적인 개입 및 중재가 필요하다[7]. 이러한 상황에서 RRT는 병동에서 발생하는 응급상황에 신속하게 대응하기 위한 체계로 간호사의 직무스트레스 및 소진을 감소시키는 데 기여하는 것으로 보고되고 있다[6].

병동 간호사는 RRT를가장 효과적으로 활용할 수 있는 주요 인력으로 간주되나[9] 실제 주도적으로 RRT를 호출한 사례는 전체 RRT 호출 중 약 11.78%에 불과한 것으로 나타나 임상 현장에서의 RRT 활용도는 여전히 미흡한 수준이다[10]. 그 배경에는 담당 주치의가 아닌 타 의료진에게 연락하는 것에 대한 심리적 저항감, 환자의 중증도와 관계없이 호출하였다는 비판에 대한 두려움, 환자 상태를 정확히 평가하는데 있어 임상적 자신감 부족, 그리고 RRT 호출이 간호업무를 가중시킨다는 부정적 인식 등으로 인해 병동 간호사들이 RRT 호출을 주저하는 것으로 알려져 있다[11,12]. 심지어 일부 간호사는 RRT를 심정지 상황에서만 사용하는 최후의 수단으로 오인하기도 하였다[13].

그럼에도 불구하고 RRT 도입 이후 병원 내 응급상황 발생 건수는 도입 이전에 비해 감소하였으며[14-16], 응급상황에서 RRT를 직접 경험한 병동 간호사일수록 RRT에 대한 만족도가 높은 것으로 보고되고 있다[17]. 즉, 응급상황 시 RRT의 신속한 대응을 통해 환자의 상태가 호전되는 경험은 간호사의 RRT에 대한 만족도를 높이며[17], 응급상황과 관련된 직무스트레스 및 소진을 감소시키는 데 기여한 것으로 나타났다[6]. 이러한 긍정적 경험은 RRT의 재이용 의지를 높이는 결과로 이어졌다[17,18]. 반면 RRT 전담간호사가 불친절하거나 병동 간호사의 의견을 존중하지 않는 태도를 보였을 경우, RRT에 대한 만족도는 낮아졌고 RRT 호출에 대한 태도도 부정적인 것으로 나타났다[17].

RRT에 관한 국외 선행연구로는 병동 간호사를 대상으로 RRT 호출 전후의 인식변화, 만족도 및 소진 감소 정도를 조사한 연구들이 보고되었으며[19,20], 응급상황에서 RRT와 함께 상황 분석 및 간호중재 수행 과정을 경험함으로써 간호사의 RRT에 대한 긍정적 인식변화에 주목한 연구들도 다수 존재한다[21,22]. 전반적으로 RRT는 간호사에게 지지자, 옹호자, 교육자 및 간호 제공자로서의 역할을 수행하며, 이에 대한 만족도는 높은 것으로 나타났다[17,23]. 또한 응급상황 발생 시 RRT의 개입으로 인해 역할 재배치와 업무재분배가 이루어지면서 병동 간호사의 소진은 감소하였다[24]. 반면 국내에서 보고된 RRT 관련 연구는 대부분 RRT의 성과를 측정하는 연구였고[5,10,25,26] 병동 간호사를 대상으로 한 RRT 관련 연구는 RRT에 대한 인식을 조사한 한편의 연구가 보고되어 있다[27]. 이 연구에서 간호사는 계층적 병원 문화와 위계적 보고 체계, RRT 호출 기준 인지 부족 등으로 RRT 호출을 망설이며 이러한 문제를 개선하기 위해 RRT에 대한 인식 제고가 필요하다 하였다.

따라서 RRT의 취지를 확산 및 정착시켜 나가기 위해 RRT에 대한 간호사의 인식과 만족도가 응급상황 관련 소진에 미치는 영향을 살펴볼 필요가 있다. 이에 본 연구에서는 병동 간호사를 대상으로 RRT에 대한 인식, 만족도 및 응급상황 관련 소진의 정도를 파악하고 응급상황 관련 소진에 영향을 미치는 요인을 확인해 보고자 한다.

### 2. 연구의 목적

본 연구는 병동 간호사의 RRT에 대한 인식 및 만족도와 응급상황 관련 소진 간의 관계를 분석하고 RRT에 대한 인식과 만족도가 응급상황 관련 소진에 미치는 영향을 파악하기 위함이며, 구체적인 목적은 다음과 같다.

1) 간호사의 RRT에 대한 인식, 만족도 및 응급상황 관련 소진 정도를 파악한다.

2) 간호사의 일반적 특성에 따른 RRT에 대한 인식, 만족도 및 응급상황 관련 소진의 차이를 파악한다.

3) 간호사의 RRT에 대한 인식, 만족도 및 응급상황 관련 소진 간의 상관관계를 파악한다.

4) 간호사의 응급상황 관련 소진에 영향을 미치는 요인을 파악한다.

## 연구 방법

### 1. 연구 설계

본 연구는 RRT에 대한 병동 간호사의 인식과 만족도가 응급상황 관련 소진에 미치는 영향을 파악하기 위한 서술적 조사연구이다.

### 2. 연구 대상

대구광역시에 소재한 계명대학교 동산병원의 일반병동에서 6개월 이상 근무한 간호사를 대상으로 하였다. 연구의 일관성을 유지하고 보건복지부 신속대응시스템 시범사업 지침[28]을 준수하기 위해 응급실, 중환자실, 소아청소년과 병동에 근무하는 간호사는 제외하였다. 또한, 6개월 미만의 신규 간호사는 배제하였는데, 이는 초보자 단계의 간호사가 응급상황을 판단하고 적절한 행동을 선택하는 데 어려움을 겪을 수 있다는 선행연구를 반영한 것이다[29]. 표본 크기는 G*Power 3.1.9.7 프로그램을 이용하여 선행연구[6]를 바탕으로 중간효과크기 .10, 유의수준 .05, 검정력 .90, 일반적 특성 및 연구변수 15개를 고려한 다중회귀분석을 기반으로 산정하였다. 그 결과 필요한 표본수는 249명이었고 탈락율 10%를 고려하여 총 277명을 연구대상자로 선정하였다. 응답이 불충분하거나 참여 철회를 원하는 대상자는 없어 총 277명의 응답을 최종 분석에 사용하였다.

### 3. 연구 도구

#### 1) RRT에 대한 인식

RRT에 대한 인식은 Jones 등[30]의 연구에서 개발된 RRT에 대한 간호사의 태도(Nurses attitudes to the medical emergency team) 측정도구를 기반으로 하였다. 해당 도구는 이후 호주보건안전위원회(Australian commission on safety and quality in health care, 2011)에 의해 24개의 문항으로 수정되었으며, 원저자에게 사용승인을 받은 후 본 연구에 사용하였다. Min [27]의 ‘조기대응팀에 대한 일반병동 간호사의 인식’ 연구에서 최초 번역되어 사용되었으며 본 연구에서 문항의 명확성과 구체성을 높이기 위해 재번역과정을 거쳤다. 재번역한 도구는 내과학 교수 1인, 간호학 교수 3인, RRT 전담간호사 3인을 대상으로 내용타당도 검증을 시행하였으며 그 결과 모든 문항의 내용타당도 지수가 .80 이상으로 확인되었다. 최종 24문항으로 구성된 도구는 Likert 5점 척도로 ‘전혀 그렇지 않다’ 1점부터 ‘매우 그렇다’ 5점까지 응답하도록 하였고, 6개의 부정 문항은 역으로 환산하여 처리하였다. 총점이 높을수록 RRT에 대한 인식이 긍정적인 것으로 해석된다. 해당 도구의 신뢰도는 선행연구에서 명확히 보고되지 않았으나, 본 연구에서는 Cronbach’s *α* = .85이었다.

#### 2) RRT에 대한 만족도

RRT에 대한 만족도는 Metcalf 등[31]의 연구에서 RRT에 대한 만족도를 측정하기 위해 개발된 도구를 원저자에게 사용승인을 받은 후 본 연구에서 번역하여 사용하였다. 번역한 도구는 내과학 교수 1인, 간호학 교수 3인, RRT 전담간호사 3인을 대상으로 내용타당도 검증을 시행하였고, 그 결과 모든 문항의 내용타당도 지수가 .80 이상으로 확인되었다. 본 도구는 총 9문항으로 구성되었으며 ‘전혀 그렇지 않다’ 1점에서 ‘매우 그렇다’ 5점까지의 5점 Likert 척도로 측정하였으며, 점수가 높을수록 RRT에 대한 만족도가 높은 것을 의미한다. 선행연구에서 본 도구의 신뢰도는 명확히 제시되어 있지 않았으나, 본 연구에서 신뢰도는 Cronbach’s *α* = .91이었다.

#### 3) 응급상황 관련 소진

응급상황 관련 소진은 Eom [6]이 응급관련 간호사의 소진 정도를 측정하기 위해 개발한 도구를 원저자에게 사용승인을 받아 사용하였으며 도구는 총 18문항, 2개의 하위영역으로 구성되었다. 극도의 탈진 9문항, 개인 성취감 9문항으로 ‘전혀 그렇지 않다’ 1점부터 ‘매우 그렇다’ 5점까지 5점 Likert 척도로 응답하도록 하였고, 7개의 긍정 문항은 역으로 환산하여 처리하였다. 총점이 높을수록 응급상황 관련 소진의 정도가 높은 것을 의미한다. 도구 개발 당시 신뢰도는 Eom [6]의 연구에서 Cronbach’s *α* = .84이었고 본 연구에서 신뢰도는 Cronbach’s *α* = .85이었다.

### 4. 자료 수집

본 연구의 자료수집은 대구광역시에 소재한 계명대학교 동산병원의 일반병동에서 근무하는 간호사를 대상으로 2023년 9월 22일에서 10월 2일까지 이루어졌다. 연구자가 병원 간호부를 직접 방문하여 연구 목적과 취지를 설명하고 자료수집 협조를 받았으며 이후 각 병동 간호사 휴게실에 연구대상자 모집공고문을 2주간 게시하였다. 설문지는 자가 보고 형식으로 작성되었으며, 연구참여에 자발적으로 동의한 대상자에게 설명문과 동의서를 제공한 후, 서면동의를 받은 후 진행되었다. 작성한 설문지는 개별봉투에 밀봉하여 자료수집이 끝난 후 연구자가 일괄 회수하였다. 설문지 작성에는 20여분 소요되었으며 설문 응답을 완료한 대상자들에게는 소정의 답례품을 제공하였다.

### 5. 자료 분석

수집된 자료는 SPSS version 27.0 (IBM Corp., Armonk, NY, USA) 프로그램을 이용하여 분석하였고, 각 항목에 대한 구체적인 분석 방법은 다음과 같다.

1) 일반적 특성은 빈도, 백분율, 평균, 표준편차로 분석하였다.

2) RRT에 대한 인식, 만족도 및 응급상황 관련 소진 정도는 평균과 표준편차로 분석하였다.

3) 일반적 특성에 따른 RRT에 대한 인식, 만족도 및 응급상황 관련 소진의 차이는 독립 t-검정과 일원분산분석 방법을 이용하였고, 사후 검증은 Scheffe 검증을 시행하였다.

4) RRT에 대한 인식, 만족도 및 응급상황 관련 소진 간의 상관관계는 Pearson 상관계수로 분석하였다.

5) 응급상황 관련 소진에 영향을 미치는 요인을 확인하기 위하여 다중회귀분석을 이용하였다.

### 6. 윤리적 고려

본 연구는 계명대학교 생명윤리위원회(Institute of Research Board, IRB)의 연구 승인을 받고 진행하였다(IRB No. 40525-202307-HR-022-02). 연구의 취지와 목적에 관한 내용을 담은 설명문을 제공 후 자발적 참여의사를 밝힌 대상자에게 서면동의를 받고 자가보고 형식의 설문을 서면으로 시행하였다. 연구대상자 설명문에는 연구과제명, 연구 참여 절차 및 방법, 개인정보 보호 및 비밀보장, 자발적 연구 참여와 중지에 관한 내용을 기재하였으며 대상자가 참여 철회를 원할 경우 언제든지 연구 참여를 철회할 수 있음을 설명하였다. 수집된 자료는 연구자만 접근 가능한 잠금장치가 있는 서랍에 보관하였고 전자 자료는 외장 하드디스크에 암호화 및 잠금장치하였다. 자료는 연구 종료 3년 후 전문가에게 의뢰하여 복원 불가능한 방법으로 영구삭제할 것이다.

## 연구 결과

### 1. 대상자의 일반적 특성에 따른 제변수간 차이

연구대상자는 274명(98.9%)으로 대부분이 여성이고 평균 연령은 32.77 ± 9.12세로, 32세 이상이 111명(40.1%)으로 가장 많았다. 종교는 무교 181명(65.3%), 최종학력은 학사 졸업이 198명(71.5%), 직위는 일반간호사가 241명(87.0%)으로 다수를 차지하였다. 근무부서는 외과계 병동 123명(44.4%), 내과계 병동 105명(37.9%), 기타 49명(17.7%) 순이었고 평균 임상 경력은 10.21 ± 9.26년으로 5년 미만이 110명(39.7%)으로 가장 많았다. 근무 형태는 3교대가 242명(87.4%), 부서의 개방성 정도는 개방적이라는 응답이 145명(52.3%), 간호직무경험수준을 평가하는 질문에는 ‘중간이다’라고 응답한 대상자가 117명(42.2%)으로 가장 많았다. 최근 1년 이내 RRT에 관한 교육을 받은 경험이 있는 대상자는 167명(60.3%)이었고 RRT를 호출해야 하는 이상 징후들에 대해 잘 알고 있는지에 관해선 250명(90.3%)이 ‘알고 있다’라고 응답하였으며 RRT 이용 경험 유무에 관해선 172명(62.1%)이 ‘이용해보지 않았다’고 응답했다.

대상자의 일반적 특성에 따른 RRT에 대한 인식은 연령(F = 5.63, *p* = .004), 종교(t = 2.55, *p* = .011), 직위(F = 10.13, *p* < .001), 임상경력(F = 8.60, *p* < .001), 근무형태(F = 13.48, *p* < .001), 부서의 개방성 정도(F = 3.22, *p* = .041), 간호직무경험수준(F = 9.17, *p* < .001)등에서 유의한 차이를 보였고, RRT 교육을 받은 간호사(t = −3.94, *p* < .001), RRT를 호출해야 하는 이상 징후를 인지하고 있는 간호사(t = 3.83, *p* < .001), RRT를 이용한 경험이 있는 간호사(t = −5.53, *p* < .001)등에서 유의한 차이를 나타냈다.

RRT에 대한 만족도에서 차이가 나타난 일반적 특성은 임상경력(F = 3.22, *p* = .041), 부서의 개방성 정도(F = 3.58, *p* = .029), 간호직무경험수준(F = 4.34, *p* = .014)등이었고 RRT 교육을 받은 간호사(t = −3.27, *p* = .001), RRT를 호출해야 하는 이상 징후가 무엇인지 아는 간호사(t = −2.59, *p* = .010), RRT를 한 번이라도 이용해 본 경험이 있는 간호사(t = −2.45, *p* = .015)등에서 유의한 차이를 보였다.

대상자의 일반적 특성에 따른 응급상황 관련 소진을 분석한 결과, 연령(F = 4.41, *p* = .013), 최종학력(F = 4.78, *p* = .009), 직위(F = 5.38, *p* = .005), 임상경력(F = 5.24, *p* = .006), 근무형태(F = 8.97, *p* < .001), 직무 경험 수준(F = 16.15, *p* < .001)등에서 유의한 차이가 나타났다(Table 1).

### 2. 대상자의 병원 RRT에 대한 인식, 만족도 및 응급상황 관련 소진 정도

연구대상자의 RRT에 대한 인식, 만족도 및 응급상황 관련 소진의 정도는 Table 2와 같다. 대상자의 RRT에 대한 인식 정도는 5점 만점 중 3.56 ± 0.39점이었고 RRT에 대한 만족도는 5점 만점 중 3.85 ± 0.53점이었다. 응급상황 관련 소진 정도는 5점 만점 중 3.07 ± 0.45점이었다.

### 3. 대상자의 RRT에 대한 인식, 만족도 및 응급상황 관련 소진 간 상관관계

연구대상자의 RRT에 대한 인식, 만족도 및 응급상황 관련 소진 간 상관관계를 분석한 결과,대상자의 응급상황 관련 소진은 연령(r = −.17, *p* = .004)과 임상경력(r = ..17, *p* = .005)에서 유의한 음의 상관관계가 나타나 연령이 높을수록, 임상경력이 많을수록 소진 수준이 낮아지는 경향을 보였다. 또한 RRT에 대한 인식(r = −.24, *p* < .001)과 RRT에 대한 만족도(r = −.16, *p* = .008) 역시 응급상황 관련 소진과 유의한 음의 상관관계를 보였다. RRT에 대한 만족도와 인식간에는 강한 양의 상관관계(r = .65, *p* < .001)가 확인되었으며, RRT에 대한 인식은 연령(r = .27, *p* < .001) 및 임상경력(r = .26, *p* < .001)과 각각 유의한 양의 상관관계를 보이는 것으로 나타났다(Table 3).

### 4. 대상자의 응급상황 관련 소진에 영향을 미치는 영향 요인

연구대상자의 응급상황 관련 소진에 영향을 미치는 요인을 검증하기 위해 다중회귀분석을 시행하였다. 일반적 특성 중 응급상황 관련 소진에서 유의한 차이를 보인 최종학력, 직위, 임상경력, 근무형태, 간호직무경험수준과 응급상황 관련 소진에 유의한 상관관계를 보인 RRT에 대한 인식과 만족도를 변수로 투입하였고, 최종학력, 직위, 근무형태, 간호직무경험 수준은 가변수(dummy variables) 처리하였다. 한편, 연령은 응급상황 관련 소진과 유의한 차이를 보였으나 임상경력과 상관계수(r = .99, *p* < .001)가 높아 다중공선성 문제가 우려되어 두 변수 중 연구주제에 더 부합하는 임상경력을 독립변수로 포함하고 연령은 제외하였다.

본 회귀분석은 유의성이 작은 변수를 제거해 나가는 후진 제거법(backward elimination)을 사용하였으며, 그 결과 회귀모형은 적합한 것으로 나타났다(F = 2.07, *p* < .001). 모형의 설명력(adjusted R^2^)은 15%였으며 공차 한계(tolerance)는 0.23∼0.89로 0.1 이상이었고 다중공선성, 자기상관 등 회귀분석의 전제조건을 충족하였다(Durbin-Watson = 2.19, variance inflation factor = 1.13∼4.34). 대상자의 응급상황 관련 소진에 영향을 미치는 요인은 학력(β = −.24, *p* = .038), 임상경력(β = −.16, *p* = .007), 근무형태(β = .19, *p* = .016), 간호직무경험수준(β = −.26, *p* < .001), RRT에 대한 인식(β = −.16, *p* = .006)등으로 확인되었다. 구체적으로 학력이 높을수록 응급상황 관련 소진이 낮았으며 임상경력이 많을수록 소진이 감소하는 경향을 보였다. 또한 3교대 근무자는 2교대 및 상근 근무자보다 소진이 높았고, 간호직무경험 수준이 높을수록 응급상황 관련 소진이 감소하는 것으로 나타났다(Table 4).

## 논의

본 연구는 RRT에 대한 병동 간호사의 인식, 만족도 및 응급상황 관련 소진의 정도를 파악하고 응급상황 관련 소진에 영향을 미치는 요인을 규명하고자 시도된 연구로 주요 연구 결과를 중심으로 다음과 같이 논의하고자 한다.

본 연구대상자의 RRT에 대한 인식은 5점 만점에 3.56 ± 0.39점으로 선행연구에서 보고한 수준과 유사한 것으로 나타났으나[13,27,32] 일부 연구에서 보고한 4점보다는 낮았다[33]. 이러한 차이는 Brown 등 [33]의 연구에서 RRT는 24시간 운영된 반면, 본 연구대상 병원에서의 운영시간은 8시간에 불과하여 RRT 운영의 이해 정도나 경험 수준이 상대적으로 낮기 때문으로 보인다. 한가지 주목할 사실은 본 연구에서의 RRT 인식점수가 2018년에 국내에서 보고된 인식점수와 유사한 것으로 나타나[27], RRT 도입 후 상당한 시간이 지났음에도 불구하고 임상 현장에서 RRT에 대한 간호사의 인식 정도에 변화가 거의 없다는 점이다. RRT에 대한 긍정적인 인식은 RRT 활용을 촉진하고 잠재적인 중증 급성기 환자 발생을 예방하는데 기여할 수 있다[11]. 뿐만 아니라 RRT를 긍정적으로 인식할수록 간호사의 자신감이 향상된다는 점을 감안할 때[24] 여전히 낮은 수준에 그치고 있는 RRT에 대한 인식을 향상시킬 수 있도록 병동 간호사들이 RRT의 도움을 받은 경험을 공유할 수 있는 피드백 시스템을 구축하는 등의 환경조성이 필요하다.

본 연구대상자의 RRT에 대한 만족도는 5점 만점에 3.85 ± 0.53점으로 동일한 도구를 사용하여 병동 간호사를 대상으로 측정하여 보고한 Metcalf 등[31]의 만족도 점수 4.63점과, 측정 도구는 다르지만 Al Qahtani [18]가 보고한 병동 간호사의 RRT에 대한 만족도 점수 수준보다 낮은 것으로 나타났다. 이러한 차이는 두 선행연구에서 RRT를 실제로 이용해 본 간호사의 비율이 각각 67%, 86.7%로, 본 연구의 37.9%보다 높았던 점과 관련이 있는 것으로 해석될 수 있다[18,31]. 즉, RRT를 이용해 본 경험이 있는 경우, RRT에 대한 만족도가 높고 재이용 의사가 높다는 사실로 짐작해 볼 때[17,18] RRT 활용의 확산을 위해 간호사로 하여금 RRT 호출을 한 번이라도 해 보도록 하는 유인책이 필요해 보인다. Al Qahtani [18]의 연구에서도 RRT의 적극적인 활용을 통한 만족도 향상을 위해 RRT를 많이 이용하는 부서에 별도의 인센티브를 제공하는 등 경영진의 경제적 지원이 뒷받침되었으며 그 결과 실제로 만족도가 높게 나온 것으로 생각된다. 또한 응급상황 발생 시 RRT 호출이 적시에 이루어져 응급상황이 해결되었을 경우 간호사는 의사결정의 자율성과 환자 안정을 위한 주체자로서 치료에 참여했다는 만족감을 경험하게 되어[24] 간호업무를 능동적으로 수행하고 업무에 대한 확신을 가지게 된다[34].

본 연구에서 응급상황 관련 소진의 정도는 3.07 ± 0.45점으로 나타났다. 이러한 결과는 선행연구에서 보고된 병동 간호사의 응급상황 관련 소진 정도 점수 3.24점[6]과 중환자실 및 응급실 간호사의 응급상황 관련 소진 정도 점수 3.03∼3.28점 보다 약간 낮거나 유사한 수준으로 나타났다[35]. 이는 병동 간호사도 응급상황으로 인한 스트레스 및 소진을 경험하는 것으로 보인다. 다만, 병동은 응급실이나 중환자실에 비해 응급상황 발생 빈도가 상대적으로 낮아 응급상황 관련 소진 정도가 다소 낮게 나타났으나, 예기치 못한 상황에서 신속한 판단 및 인지의 어려움, 당혹감 등으로 인해[36] 잠재적으로 높은 수준의 소진을 경험할 가능성이 존재한다. 더불어 심정지와 같이 예측이 어려운 응급상황은 업무 지연을 야기시키고, 이로 인해 간호사는 신체적 소진과 무력감을 경험하게 되며 소진의 정도가 심할 경우 스스로에 대한 자기 비하나 부정적인 자기 평가로 이어질 수 있다[37]. 따라서 응급상황 관련 소진 감소를 위한 인력 지원체계로 RRT를 적극적으로 활용할 수 있는 실질적인 지원방안과 중재프로그램의 도입이 요구된다.

본 연구에서 응급상황 관련 소진은 연령, 임상경력, RRT에 대한 인식, RRT에 대한 만족도와 유의한 부적 상관관계를 보였다. 즉, 병동 간호사의 연령과 임상경력이 많을수록 RRT에 대한 인식과 만족도가 높을수록 응급상황 관련 소진이 낮아지는 경향을 보였다. 이러한 변수들을 포함하여 다중회귀분석을 실시한 결과, 학력, 임상경력, 근무형태, 간호직무경험 수준, RRT에 대한 인식이 응급상황 관련 소진에 유의한 영향을 미치는 요인으로 확인되었다.

학력이 높을수록 응급상황 관련 소진이 낮은 경향을 보였는데 이는 고학력 간호사가 보다 효과적인 스트레스 대처전략을 활용하고, 전문적인 지식과 경험을 통해 업무에 대한 자신감을 보유할 가능성이 높기 때문으로 해석된다. 기존 연구에서도 고학력 간호사가 낮은 소진을 보인다는 연구 결과가 보고된 바 있다[7]. 또한 임상경력이 많을수록, 그리고 간호직무경험 수준이 전문가 단계인 간호사일수록 응급상황 관련 소진이 감소하는 경향을 보였다. 이는 다양한 임상경험을 통해 업무 숙련도가 향상되어 동일한 스트레스 상황에서도 유연한 대처가 가능하며, 심리적 회복 탄력성이 향상되어 스트레스를 보다 효과적으로 관리할 수 있기 때문으로 해석된다[6]. 이러한 결과는 임상경험이 부족한 간호사가 응급상황에 침착하게 대처할 수 있는 능력이 미흡하고 이에 따른 부담과 불안감이 증가하여 더 높은 소진을 경험한다는 선행연구 결과와도 일치한다[12]. 특히, 신규 간호사일수록 업무 우선순위 파악 및 환자의 생리적 변화를 평가하는 데 두려움을 느끼고 의사결정에 대한 불안감으로 인해 선임간호사보다 더 높은 소진을 경험하는 것으로 나타났다[38,39]. 즉, 직무능력이 향상될수록 응급상황에서 느끼는 부담감이 줄어들고, 결과적으로 소진 예방에 긍정적인 영향을 미칠 수 있을 것이다. 근무형태에 있어서는 3교대 근무자가 2교대 또는 상근근무자보다 응급상황 관련 소진이 유의하게 높았다. 이는 교대근무가 신체리듬을 변화시키고, 수면부족 및 피로누적 등으로 인해 신체적·정신적 스트레스를 증가시키는 요인이 될 수 있다는 기존 연구와 유사하다[37].

본 연구에서 RRT에 대한 병동 간호사의 인식이 응급상황 관련 소진을 감소시키는 중요한 요인임을 확인하였다. 선행연구에서도 병동 간호사는 응급상황에서 RRT를 불안정한 환자 관리를 위한 자산으로 인식하고[40] RRT의 신속한 개입이 환자의 예후를 개선할 뿐만 아니라 자신의 업무 부담을 완화시키는데 도움이 된다고 평가하는 것과[17] 유사한 결과라 할 것이다. 특히, RRT에 대한 인식이 긍정적인 간호사일수록 RRT를 적극적으로 활용하게 되며, 이를 통해 응급상황에서 보다 능동적으로 대처할 수 있게 되고[24] 이러한 경험이 반복될수록 병동 간호사의 직무 스트레스가 감소하고 소진 완화에도 긍정적인 영향을 미치는 것으로 나타났다[6,23]. 반면, RRT를 부정적으로 인식하는 간호사는 RRT 호출을 주저하거나 비효율적인 의사소통으로 인해 응급상황에서 더 높은 소진을 경험하기에[11,12] RRT에 대한 인식 개선과 교육프로그램 강화는 병동 간호사의 소진을 줄이는 중요한 전략이 될 수 있다.

다만, 본 연구에서 응급상황 관련 소진에 대한 전체 설명력이 15%에 그쳐 RRT 인식이 소진에 영향을 미친다는 방향성은 확인하였지만, 문헌에서 보고된 것처럼 영향력의 정도는 높지 않음을 알 수 있었다. 이러한 결과는 응급상황 관련 소진이 단순히 RRT에 대한 인식과 만족도 뿐만 아니라 개인적, 조직적, 환경적 요인 등 다양한 요인에 의해 영향을 받는 복합적인 문제일 수 있음을 시사한다. 또한 본 연구 대상 병원은 RRT 시범사업 군별 특성상 3군에 해당되어 RRT를 하루 8시간만 운영하고 있었으므로 대부분 24시간 운영하는 병원을 대상으로 한 연구결과와 차이가 있는 것으로 보인다. 따라서 RRT 운영의 특성을 고려한 추후 연구를 통해 RRT에 대한 인식과 소진의 관련성을 탐색해 나갈 필요가 있다.

이상의 연구 결과를 바탕으로, 본 연구는 병동 간호사의 RRT에 대한 인식, 만족도 및 응급상황 관련 소진의 정도를 파악하고 이들의 관계를 살펴 보았다. 그 결과 RRT의 인식과 만족도는 모두 응급상황 관련 소진과 부적 관계를 보였으며, 특히 이 중 RRT에 대한 인식은 소진에 영향을 미치는 주요 요인 중 하나로 나타났다. RRT를 긍정적으로 인식하고 적극적으로 활용하는 것은 병동 간호사의 소진을 줄이고 업무 만족도를 향상시키며, 환자 안전을 강화하는 데 기여할 것이다. 또한 본 연구는 국내 임상간호사를 대상으로 RRT에 대한 인식과 만족도가 응급상황 관련 소진에 미치는 영향을 처음으로 분석하였다는 점에서 의의가 있다. 이는 국내 의료 환경에서 RRT 활용의 중요성을 재확인하고, 간호사의 소진을 줄이기 위한 효과적인 전략을 마련하는 데 근거를 제공한다는 점에서 의미가 크다.

본 연구의 제한점으로 첫째, 본 연구 대상자는 광역시 소재의 한 개 상급종합병원에서 근무하는 병동 간호사를 대상으로 수행되었으므로 연구 결과를 일반화하는데 제한점이 있다. 둘째, RRT 운영시간이 다양한 병원의 간호사를 대상으로 RRT 인식 및 만족도와 소진 간의 관계를 비교 분석할 필요가 있다. 셋째, 본 연구에서는 국내에서 개발된 RRT 관련 인식 및 만족도 측정 도구가 없어 국외에서 사용된 도구를 번역·활용하였으며, 이로 인해 일부 문항이 국내 임상 환경과 부합하지 않을 가능성이 있다. 따라서 향후 국내 실정에 적합한 타당한 측정 도구의 개발이 필요하다. 넷째, 응급상황 관련 소진이 다양한 개인적, 조직적, 환경적 요인에 의해 복합적으로 영향을 받을 수 있음을 고려하여 소진 완화를 위한 시뮬레이션 교육 등 응급역량 강화 교육프로그램 개발이 필요하다.

## 결론

본 연구에서 간호사를 대상으로 RRT에 대한 인식, 만족도 및 응급상황 관련 소진 간의 관계를 파악하고 응급상황 관련 소진에 영향을 미치는 요인을 파악하고자 하였다. 연구 결과 RRT에 대한 인식과 만족도가 높을수록 응급상황 관련 소진이 감소하는 것으로 나타났으며 이 중 RRT에 대한 인식은 병동 간호사의 응급상황 관련 소진에 영향을 미치는 주요 요인으로 확인되었다. 따라서 RRT의 적극적인 활용과 인식 개선이 간호사의 소진 관리에 중요한 전략임을 알 수 있었다. 본 연구 결과를 바탕으로 교육프로그램 개선, RRT 활용 증진, 정책적 지원 강화 등의 전략을 적극적으로 추진할 필요가 있다. 본 연구결과를 바탕으로 다음과 같이 제언하고자 한다.

첫째, RRT에 대한 국내연구 대다수가 RRT의 성과에 관한 연구로 국내 실정에 적합한 RRT에 대한 인식 및 만족도 도구 개발을 제언한다.

둘째, 간호환경의 특성을 고려하여 RRT 활용을 증진시키는 요인과 저해 요인을 규명하고, 이를 기반으로 효율적인 RRT 운영체계 구축과 병동 간호사와의 협업 강화를 위한 제도적 기반 마련을 제언한다.

셋째, 응급상황과 관련된 간호사의 소진을 예방하고 임상 대응능력을 향상시키기 위해, 실제 상황을 반영한 시뮬레이션 기반의 응급역량 강화 교육프로그램의 체계적 개발을 제언한다.

## Figures and Tables

**Table 1.** Differences among Variables Based on General Characteristics (N = 277)

Variables	Categories		Perception of the RRT		Satisfaction with the RRT		Burnout related to emergency situations	
			t or F (*p*) Scheffè		t or F (*p*) Scheffè		t or F (*p*) Scheffè	
Sex	Women	274 ( 98.9)	3.56 ± 0.39	0.32 (.353)	3.85 ± 0.52	0.85 (.395)	3.08 ± 0.44	−1.93 (.054)
	Men	3 (1.1)	3.76 ± 0.39		4.11 ± 0.77		2.57 ± 0.45	
Age (yr)	≤ 26^a^	81 (29.2)	3.47 ± 0.30	5.63 (.004)	3.85 ± 0.46	1.04 (.354)	3.19 ± 0.35	4.41 (.013)
	27∼31^b^	85 (30.7)	3.52 ± 0.35		3.79 ± 0.62		3.06 ± 0.49	
	≥ 32^c^	111 (40.1)	3.65 ± 0.45	a < c	3.91 ± 0.49		2.99 ± 0.47	a > c
		32.77 ± 9.12						
Religion	Yes	96 (34.7)	3.64 ± 0.46	2.55 (.011)	3.90 ± 0.52	1.12 (.263)	3.07 ± 0.47	−0.02 (.988)
	No	181 (65.3)	3.51 ± 0.34		3.83 ± 0.53		3.07 ± 0.44	
Education level	Associate’s degree^a^	16 (5.8)	3.48 ± 0.34	1.44 (.240)	3.84 ± 0.51	0.29 (.747)	3.35 ± 0.42	4.78 (.009)
	Bachelor's degree^b^	198 (71.5)	3.54 ± 0.38		3.87 ± 0.53		3.08 ± 0.43	
	≥ Master’s degree^c^	63 (22.7)	3.64 ± 0.41		3.81 ± 0.52		2.97 ± 0.48	a > c
Current position	Staff^a^	241 (87.0)	3.52 ± 0.37	10.13 (< .001)	3.83 ± 0.53	2.46 (.087)	3.10 ± 0.43	5.38 (.005)
	Charge^b^	26 (9.4)	3.76 ± 0.39		4.01 ± 0.39		2.82 ± 0.49	
	Head^c^	10 (3.6)	3.93 ± 0.51	a < b,c	4.09 ± 0.66		2.94 ± 0.58	a > b
Department	General medicine ward	105 (37.9)	3.53 ± 0.40	1.18 (.309)	3.87 ± 0.51	0.29 (.747)	3.08 ± 0.49	0.40 (.673)
	Surgical ward	123 (44.4)	3.59 ± 0.37		3.86 ± 0.58		3.08 ± 0.44	
	Other	49 (17.7)	3.50 ± 0.41		3.80 ± 0.41		3.02 ± 0.38	
Clinical career (yr)	< 5^a^	110 (39.7)	3.50 ± 0.34	8.60 (< .001)	3.87 ± 0.50	3.22 (.041)	3.14 ± 0.42	5.24 (.006)
	5∼< 10^b^	67 (24.2)	3.47 ± 0.31		3.72 ± 0.58		3.13 ± 0.44	
	≥ 10^c^	100 (36.1)	3.68 ± 0.46	a,b < c	3.94 ± 0.51	b < c	2.96 ± 0.47	a,b > c
		10.21 ± 9.26						
Work shifts	Three shifts^a^	242 (87.4)	3.52 ± 0.37	13.48 (< .001)	3.83 ± 0.52	2.31 (.101)	3.11 ± 0.43	8.97 (< .001)
	Two shifts^b^	23 (8.3)	3.86 ± 0.40		4.05 ± 0.50		2.72 ± 0.49	
	Full-time day shift^c^	12 (4.3)	3.87 ± 0.48	a < b,c	3.98 ± 0.67		2.94 ± 0.54	a>b
Department openness	Low openness^a^	41 (14.8)	3.49 ± 0.38	3.22 (.041)	3.91 ± 0.46	3.58 (.029)	3.16 ± 0.54	2.04 (.133)
	moderate openness^b^	91 (32.9)	3.50 ± 0.34		3.73 ± 0.53		3.11 ± 0.47	
	High openness^c^	145 (52.3)	3.61 ± 0.41	-	3.91 ± 0.53	b < c	3.02 ± 0.41	
Level of nursing job experience	Novice^a^	73 (26.4)	3.43 ± 0.37	9.17 (< .001)	3.78 ± 0.55	4.34 (.014)	3.23 ± 0.42	16.15 (< .001)
	Intermediate^b^	117 (42.2)	3.54 ± 0.31		3.81 ± 0.49		3.12 ± 0.40	
	Expert^c^	87 (31.4)	3.69 ± 0.46	a,b<c	3.99 ± 0.54	a,b < c	2.86 ± 0.47	a.b > c
RRT education experience	Yes	167 (60.3)	3.63 ± 0.39	−3.94 (< .001)	3.94 ± 0.51	−3.27 (.001)	3.06 ± 0.43	0.48 (.634)
	No	110 (39.7)	3.45 ± 0.37		3.73 ± 0.53		3.09 ± 0.48	
Recognition of RRT activation criteria	Yes	250 (90.3)	3.58 ± 0.38	3.83 (< .001)	3.88 ± 0.52	−2.59 (.010)	3.06 ± 0.45	1.20 (.230)
	No	27 (9.7)	3.29 ± 0.33		3.61 ± 0.56		3.17 ± 0.43	
Experience of using RRT	Yes	105 (37.9)	3.46 ± 0.37	−5.53 (< .001)	3.95 ± 0.52	−2.45 (.015)	3.04 ± 0.47	0.98 (.325)
	No	172 (62.1)	3.71 ± 0.37		3.79 ± 0.53		3.09 ± 0.44	

Values are presented as the mean ± standard deviation or n (%). RRT = Rapid response team.

**Table 2.** Level of Perceptions of the RRT, Satisfaction with the RRT, and Burnout Related to Emergency Situations (N = 277)

Variables	Range	M ± SD	Min	Max
Perception of RRT	1~5	3.56 ± 0.39	2.38	5.00
Satisfaction of RRT	1~5	3.85 ± 0.53	2.33	5.00
Burnout related to emergency situations	1~5	3.07 ± 0.45	1.67	4.17

RRT = Rapid response team; M = Mean; SD = Standard deviation; Min = Minimum; Max = Maximum.

**Table 3.** Correlation of Perception of the RRT, Satisfaction with the RRT, and Burnout Related to Emergency Situations (N = 277)

Variables	Age	Clinical career	Perception of RRT	Satisfaction of RRT	Burnout related to emergency situations
	r (*p*)				
Clinical career	.99 (< .001)	1	-	-	-
Perception of the RRT	.27 (< .001)	.26 (< .001)	1	-	-
Satisfaction with the RRT	.09 (.117)	.09 (.134)	.65 (< .001)	1	-
Burnout related to	−.17 (.004)	−.17 (.005)	−.24 (< .001)	−.16 (.008)	1
emergency situations					

RRT = Rapid response team.

**Table 4.** Factors Affecting Nurses' Burnout Related to Emergency Situations (N = 277)

Variables	B	SE	β	t	*p*
(Constant)	3.62	.31		11.84	< .001
Education level^†^					
Bachelor's degree	−0.21	.11	−.21	−1.90	.058
≥ Master's degree	−0.26	.12	−.24	−2.08	.038
Current position^‡^					
Charge	−0.22	.11	−.14	−1.94	.054
Clinical career (yr)	−0.01	.01	−.16	−2.74	.007
Work shifts^§^					
Three shifts	0.26	.11	.19	2.43	.016
Level of nursing job experience^∥^					
Expert	−0.25	.06	−.26	−3.95	< .001
Perception of the RRT	−0.18	.07	−.16	−2.76	.006
Durbin-Watson = 2.19, R^2^ (adj R^2^) = .18 (.15), F = 2.07, *p * < .001					

SE = Standard error; RRT = Rapid response team.

^†^Education level (reference = associate’s degree); ^‡^Current position (reference = head nurse); ^§^Work Shift (reference = full-time day shift); ∥Level of nursing job experience (reference = novice).

## Data Availability

Please contact the corresponding author for data availability.
